# Supplementary material for: Effect of Combined Bee Venom Acupuncture and NSAID Treatment for Non-Specific Chronic Neck Pain: A Randomized, Assessor-Blinded, Pilot Clinical Trial
Source: Toxins (Basel). 2021 Jun 23;13(7):436. doi: 10.3390/toxins13070436 (PMC8309931; doi:10.3390/toxins13070436)
Supplement: Supplementary file 1 [file toxins-13-00436-s001.zip › toxins-1239589-supplementary/Supplement 2.pdf]

Supplementary Materials: Effect of Combined Bee Venom Acupuncture and NSAID Treatment for Non-Specific Chronic Neck Pain: A Randomized, Assessor-Blinded, Pilot Clinical Trial

Boram Lee, Byung-Kwan Seo, O-Jin Kwon, Dae-Jean Jo, Jun-Hwan Lee and Sanghun Lee

Table S1. The results of vital sign and laboratory tests.

	BVA (N=20)	NSAIDs (N=20)	Combined (N=20)
SBP (mmHg)			
Baseline	119.8 (113.5, 126.0)	120.1 (112.7, 127.4)	119.6 (112.5, 126.6)
Week 8	120.0 (115.5, 124.5)	119.1 (113.8, 124.4)	123.3 (116.9, 129.6)
Mean Difference†	0.25 (−4.58, 5.08)	−0.95 (−5.05, 3.15)	3.70 (−2.59, 9.99)
p-value††	0.9148	0.6329	0.2333
DBP (mmHg)			
Baseline	73.05 (68.91, 77.19)	74.95 (70.86, 79.04)	72.65 (67.30, 78.00)
Week 8	74.35 (70.25, 78.45)	74.70 (70.70, 78.70)	75.65 (71.92, 79.38)
Mean Difference†	1.30 (−3.16, 5.76)	−0.25 (−2.05, 1.55)	3.00 (−1.78, 7.78)
p-value††	0.5487	0.7747	0.2042
Pulse (times/minute)			
Baseline	78.40 (74.56, 82.24)	79.95 (76.46, 83.44)	74.90 (70.62, 79.18)
Week 8	80.30 (76.47, 84.13)	79.50 (75.23, 83.77)	76.35 (71.87, 80.83)
Mean Difference†	1.90 (−1.58, 5.38)	−0.45 (−4.98, 4.08)	1.45 (−2.20, 5.10)
p-value††	0.2667	0.8376	0.4155
Temperature (℃)			
Baseline	36.52 (36.48, 36.56)	36.50 (36.45, 36.55)	36.46 (36.41, 36.50)
Week 8	36.52 (36.48, 36.56)	36.50 (36.45, 36.54)	36. 47 (36.43, 36.51)
Mean Difference†	0.00 (−0.05, 0.05)	−0.01 (−0.05, 0.04)	0.02 (−0.04, 0.07)
p-value††	0.9999	0.8154	0.5620
AST			
Baseline	20.15 (21.33, 28.97)	23.65 (19.45, 27.85)	23.05 (19.29, 26.81)
Week 4	23.15 (20.13, 26.17)	22.15 (18.29, 26.01)	22.30 (19.38, 25.22)
Mean Difference†	−2.00 (−4.47, 0.47)	−15.0 (−5.48, 2.48)	−0.75 (−2.45, 0.95)
p-value††	0.1067	0.4394	0.3684
ALT			
Baseline	22.10 (17.38, 26.82)	19.80 (13.46, 26.14)	18.95 (12.57, 25.33)
Week 4	20.65 (16.61, 24.69)	19.00 (12.51, 25.49)	17.35 (12.29, 22.41)
Mean Difference†	−1.45 (−4.34, 1.44)	−0.80 (−2.32, 0.72)	−1.60 (−4.29, 1.09)
p-value††	0.3076	0.2830	0.2280
BUN			
Baseline	16.15 (13.52, 18.78)	12.55 (11.11, 13.99)	12.95 (11.68, 14.22)
Week 4	16.55 (14.35, 18.75)	12.70 (11.25, 14.15)	12.05 (10.54, 13.56)
Mean Difference†	0.40 (−0.62, 1.42)	0.15 (−1.25, 1.55)	−0.90 (−2.48, 0.68)
p-value††	0.4233	0.8252	0.2471
ALP			
Baseline	219.4 (191.6, 247.2)	193.3 (165.9, 220.6)	209.7 (169.9, 249.6)

Week 4	221.6 (194.8, 248.3)	200.0 (167.5, 232.4)	220.6 (185.5, 255.6)
Mean Difference†	2.15 (−11.57, 15.87)	6.70 (−6.87, 20.27)	10.82 (−8.20, 29.83)
p-value††	0.7464	0.3146	0.2486
Total bilirubin			
Baseline	0.645 (0.508, 0.782)	0.605 (0.506, 0.704)	0.630 (0.518, 0.741)
Week 4	0.610 (0.493, 0.727)	0.550 (0.485, 0.615)	0.665 (0.546, 0.734)
Mean Difference†	−0.035 (−0.120, 0.050)	−0.055 (−0.124, 0.014)	0.035 (−0.082, 0.152)
p-value††	0.3991	0.1102	0.5384
Creatinine			
Baseline	0.900 (0.830, 0.970)	0.830 (0.762, 0.898)	0.810 (0.742, 0.878)
Week 4	0.905 (0.823, 0.987)	0.825 (0.759, 0.891)	0.830 (0.754, 0.906)
Mean Difference†	0.005 (−0.037, 0.047)	−0.005 (−0.044, 0.034)	0.020 (−0.016, 0.056)
p-value††	0.8037	0.7894	0.2585

† Mean difference from baseline †† paired t-test.
